# Prediction of delayed breastfeeding initiation among mothers having children less than 2 months of age in East Africa: application of machine learning algorithms

**DOI:** 10.3389/fpubh.2024.1413090

**Published:** 2024-09-02

**Authors:** Agmasie Damtew Walle, Zenebe Abebe Gebreegziabher, Habtamu Setegn Ngusie, Sisay Yitayih Kassie, Abera Lambebo, Fitsum Zekarias, Tadesse Mamo Dejene, Shimels Derso Kebede

**Affiliations:** ^1^Department of Health Informatics, School of Public Health, Debre Berhan University, Debre Birhan, Ethiopia; ^2^Department of Epidemiology and Biostatistics, School of Public Health, Debre Berhan University, Debre Birhan, Ethiopia; ^3^Department of Health Informatics, School of Public Health, College of Medicine and Health Sciences, Woldia University, Woldia, Ethiopia; ^4^Department of Health Informatics, School of Public Health, College of Medicine and Health Science, Hawassa University, Hawassa, Ethiopia; ^5^Department of Public Health, School of Public Health, Debre Berhan University, Debre Birhan, Ethiopia; ^6^Department of Health Informatics, School of Public Health, College of Medicine and Health Sciences, Wollo University, Dessie, Ethiopia

**Keywords:** machine learning, breastfeeding initiation, children, East Africa, DHS

## Abstract

**Background:**

Delayed breastfeeding initiation is a significant public health concern, and reducing the proportion of delayed breastfeeding initiation in East Africa is a key strategy for lowering the Child Mortality rate. However, there is limited evidence on this public health issue assessed using advanced models. Therefore, this study aimed to assess prediction of delayed initiation of breastfeeding initiation and associated factors among women with less than 2 months of a child in East Africa using the machine learning approach.

**Methods:**

A community-based, cross-sectional study was conducted using the most recent Demographic and Health Survey (DHS) dataset covering the years 2011 to 2021. Using statistical software (Python version 3.11), nine supervised machine learning algorithms were applied to a weighted sample of 31,640 women and assessed using performance measures. To pinpoint significant factors and predict delayed breastfeeding initiation in East Africa, this study also employed the most widely used outlines of Yufeng Guo’s steps of supervised machine learning.

**Results:**

The pooled prevalence of delayed breastfeeding initiation in East Africa was 31.33% with 95% CI (24.16–38.49). Delayed breastfeeding initiation was highest in Comoros and low in Burundi. Among the nine machine learning algorithms, the random forest model was fitted for this study. The association rule mining result revealed that home delivery, delivered by cesarean section, poor wealth status, poor access to media outlets, women aged between 35 and 49 years, and women who had distance problems accessing health facilities were associated with delayed breastfeeding initiation in East Africa.

**Conclusion:**

The prevalence of delayed breastfeeding initiation was high. The findings highlight the multifaceted nature of breastfeeding practices and the need to consider socioeconomic, healthcare, and demographic variables when addressing breastfeeding initiation timelines in the region. Policymakers and stakeholders pay attention to the significant factors and we recommend targeted interventions to improve healthcare accessibility, enhance media outreach, and support women of lower socioeconomic status. These measures can encourage timely breastfeeding initiation and address the identified factors contributing to delays across the region.

## Introduction

The World Health Organization (WHO) and the United Nations Children’s Fund (UNICEF) recommend that breastfeeding should start within the first hour after birth and it is the most important source of nutrition for infants, protecting against nearly all childhood diseases (1). Breast milk contains various proteins that function as antibodies, bolstering the body’s defenses against illnesses later in life. Additionally, it fulfills all the nutritional requirements of a newborn for the first 6 months (2, 3).

Breastfeeding is the optimal strategy for promoting child survival and development, potentially saving around 820,000 children worldwide every year through adherence to proper breastfeeding practices (1). Additionally, breastfeeding can significantly influence a child’s intelligence and cognitive development later in life. The benefits extend beyond infancy, positively impacting both the child and the mother throughout their lifetimes. Particularly, initiating breastfeeding early and maintaining exclusive breastfeeding for the first 6 months, followed by complementary feeding up to 2 years and beyond, yields numerous health advantages for both mother and child (3–6). Despite efforts by the World Health Organization (WHO) and the United Nations Children’s Fund (UNICEF) to promote early breastfeeding, more than 50% of infants worldwide still do not start breastfeeding within the recommended first hour of life. Additionally, if early initiation of breastfeeding within the recommended time and exclusive breastfeeding are widely implemented (7). In the United States of America study concluded that early breastfeeding initiation has a strong association with reducing post-perinatal infant mortality which means delayed initiation of breastfeeding increases infant and neonatal mortality (8).

Globally, the delayed initiation of breastfeeding is linked to higher mortality rates among children under five and neonates. In 2019, the under-five mortality rate was 37.7 per 1,000 live births, resulting in 5.2 million children dying before their fifth birthday. Similarly, the neonatal mortality rate was 17.7 per 1,000 live births, leading to 2.4 million neonatal deaths that same year. The Sustainable Development Goal aims to reduce neonatal mortality to 12 per 1,000 live births by 2030. However, 63 countries are projected to miss this target, despite 116 countries making significant progress. Thus, it is crucial to focus on reducing neonatal, infant, and child mortality rates to prevent an estimated 48.1 million under-five deaths from 2020 to 2030 (9–11).

A global study on delayed initiation of breastfeeding found varying prevalence rates in different regions. In 35 sub-Saharan African countries, the prevalence was 77.0% (12), In Odisha, India, it was 63.6% (13), while health surveys in 58 low-and middle-income countries reported a rate of 53.8% (14), In Ghana, the prevalence was 44.9% (15), and evidence from the 2017/2018 Benin demographic and health survey showed 44.0% (16), Sub-Saharan Africa data using demographic and health surveys indicated a 43.0% prevalence (17), whereas Tigray in Northern Ethiopia reported 40.0% (18), In Abu Dhabi, United Arab Emirates, the prevalence was 35.8% (19), Kilimanjaro region in northern Tanzania showed 28.9% (20), and Ethiopia overall had a prevalence of 24.3% (21), and the Moshi municipal area in northern Tanzania had the lowest prevalence at 14.1% (1).

Neonatal and child mortality are significant challenges in sub-Saharan Africa, posing major obstacles to achieving the Sustainable Development Goals (SDGs). This region is crucial for the success of global health initiatives, particularly universal health coverage because it accounts for a substantial proportion of child and neonatal deaths worldwide. The estimated economic cost of under-five mortality in sub-Saharan Africa is projected to reach $42 billion by 2030. Addressing these mortality rates is essential for making progress toward universal health targets and improving global health outcomes (22–24). Delayed initiation of breastfeeding is a significant challenge in sub-Saharan African countries and research indicates that most mothers in this region do not start breastfeeding within 1 h of their baby’s birth. This delay is influenced by various maternal and neonatal factors (25–27).

Delayed initiation of breastfeeding has serious and potentially fatal consequences. Research shows that when mothers delay breastfeeding, regardless of the cause, it directly affects neonatal mortality, infant mortality, and child survival. Additionally, delaying the start of breastfeeding can increase the risk of early childhood obesity, meaning that children who are not breastfed early may be more prone to developing obesity in their early years (28–33).

Previous studies have identified several factors contributing to the delayed initiation of breastfeeding, defined as starting breastfeeding after the first hour of life. One of the primary factors is the mode of delivery. Most research articles indicate that cesarean delivery is a significant contributor to delays in the initiation of breastfeeding (14, 17, 19, 21, 25–27, 34–38). Moreover, place of delivery (14, 17, 21, 25, 26, 35, 36, 38), media exposure (16, 17, 25, 26), frequent ANC visits (21, 35–37), maternal education (18, 26, 34, 38), marital status (25, 27, 34, 37), breastfeeding information (1, 18, 34), maternal age (19, 26, 36), wealth index (17, 26), skin to skin contact (17, 39), parity (18, 25, 26, 34), women occupation (40), religion (41) were significant association with delayed initiation of breastfeeding.

To our knowledge, there is limited research on breastfeeding initiation using advanced models in East Africa, where maternal, child, infant, and neonatal mortality rates are alarmingly high. Therefore, it is essential to apply advanced machine learning techniques to predict delayed breastfeeding initiation and pinpoint the contributing factors among mothers with children under 2 years old. Machine learning is particularly valuable for this study because it can handle complex and large datasets, revealing patterns and relationships that traditional methods might miss. This approach allows for a thorough analysis of multiple variables and interactions, providing precise and actionable insights. By using machine learning, we can address the existing evidence gap and offer reliable data to guide effective interventions, ultimately improving decision-making both locally and within the wider scientific community.

## Materials and methods

### Study design and study setting

A community-based cross-sectional study was conducted in the East African region from 2011 to 2021 using recent Demographic and Health Surveys (DHS) data. Among 19 East African countries (Burundi, Comoros, Djibouti, Ethiopia, Eritrea, Kenya, Madagascar, Malawi, Mauritius, Mozambique, Rwanda, Seychelles, Somalia, Tanzania, Uganda, Zambia, South Sudan, Zimbabwe, and Sudan),14 countries had available Demographic and Health Surveys (DHS) datasets, while 5 countries (Djibouti, Somalia, South Sudan, Seychelles, and Mauritius) did not have dataset. From the 14 countries with data, Madagascar, Eritrea, and Sudan were excluded from the analysis due to outdated datasets and data restrictions (42). Finally, to be more representative of East Africa, this study used recent standard DHS data from 11 countries (Burundi, Ethiopia, Comoros, Uganda, Rwanda, Tanzania, Mozambique, Zimbabwe, Kenya, Zambia, and Malawi).

### Data source, study population, and sampling technique

Demographic and Health Surveys (DHS)-based secondary data analysis was used to carry out the study. Each country’s survey has a variety of datasets; for this analysis, we selected the Individual Record (IR) file. These datasets include those for males, women, children, births, individuals, and households. Demographic and Health Surveys (DHS)-employed a two-stage stratified cluster sampling technique, employing the Population and Housing Census (PHC) as the sampling frame. Enumeration Areas (EAs) were selected in the first stage using independent selection in each sampling stratum and probability sampling proportionate to the size of the EAs. The second phase involved the methodical selection of households. A thorough sampling process was given in the complete DHS report (43, 44). After managing the data a total weighted sample of 31,640 respondents was included in the study for further analysis.

### Study variables

The outcome variable for this study was delayed breastfeeding initiation, which is described as women who failed to initiate breastfeeding within 1 h after birth as per WHO recommendation (36). The outcome variable is dichotomized as “1” for women who start breastfeeding after 1 h (delayed initiation) and “0” for starting breastfeeding within 1 h (timely initiation). Whereas, the independent variables for this study were residence, maternal age, women’s educational level, marital status, religion, wealth index, media exposure, women’s occupation, place of delivery, parity, number of ANC visits, birth interval, sex of household head, husband education level, distance to a health facility, mode of delivery, sex of the child, and type of childbirth.

### Data management and analysis

To restore the representativeness of the survey and take the sampling design into account for precise statistical estimations, the data were weighted using the primary sampling unit, sampling weight, and strata before performing the statistical analysis.

Using STATA software version 17, the actual samples containing those variables that were selected were taken out of the DHS measures and exported to a CSV file. For further analysis, the data was then imported into a Jupyter Notebook version 3.11. Preprocessing techniques include feature selection, data discretization, and outlier detection, KNN imputation for missing value management, explanatory data analysis, and target feature balance. Records and features were used for model construction with 80% training and 20% testing data once data preparation processes were completed.

Supervised machine learning algorithms such as Random Forest, Ada Boost, Gaussian NB, MLP, Decision Tree, Logistic Regression (LR), random forest (RF), K-Nearest Neighbors (KNN), Extreme Gradient Boosting (XG Boost), support vector machines (SVM) (45–48), was performed to predict delayed breastfeeding initiation among reproductive age women in East Africa.

**Figure 1:**
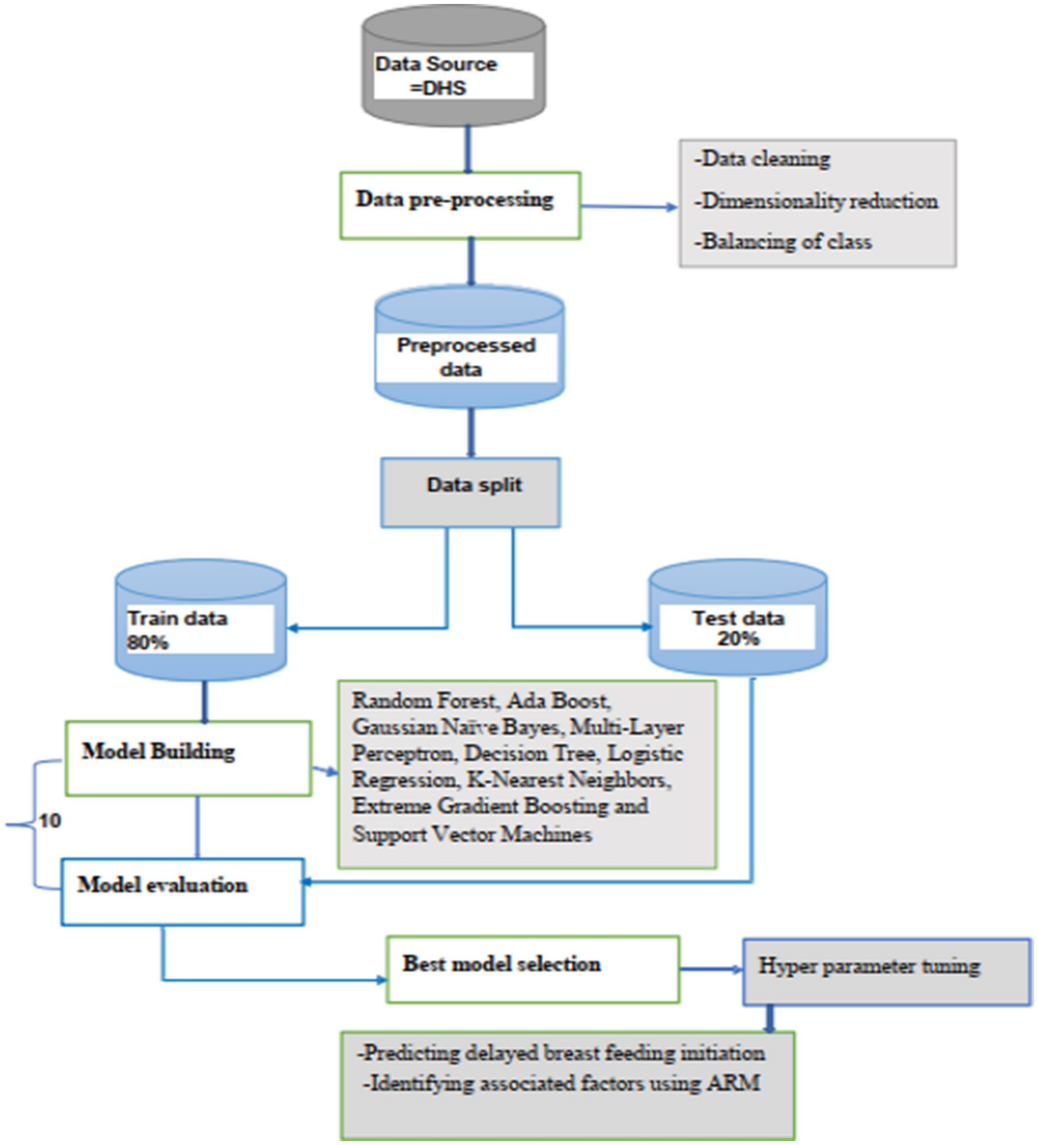
Overview flow chart of methodologies.

A tenfold cross-validation method was used for training the models and confusion matrix, and the receiver operating area under the curve was applied to evaluate the performance of the model. After hyperparameter tuning of the best-performed model then trained with balanced data for the final prediction to show unseen patterns in data. The feature importance method was used to explore the relationship between the predictors and the outcome variable using random forest. Moreover, the association rule mining technique is also employed to discover how features are associated with each other in individuals. Finally, the overall methodology workflow is shown in Figure 1.

### Ethical consideration

This study utilized secondary data, which was accessed through an online request to http://www.dhsprogram.com. A consent letter was obtained from the Demographic and Health Surveys (DHS) Program. The data used did not contain any personally identifiable information and was publicly available. The DHS public-use datasets, approved by the IRB, ensure that respondents, families, and sample communities cannot be identified. The data files exclude household addresses and names, and geographic identifiers only reach the regional level, which typically covers broad areas encompassing multiple states or provinces.

## Results

### Socio-demographic and economic characteristics of the study participants

The mean age of the participant was 27.6 ± (0 0.04 SD). About three-fourths, 24,155 (76.3%) of the study participants were from rural residents and half of the women, 15,854 (50.1%) attained primary education. 14,450 (45.6%) of them had poor wealth status. 20,351 (64.3%) of them had media exposure and nearly half, 15,955 (50.4%) of the participants had above four ANC visits. 24,464 (77.3%) of the participants delivered at a health facility and 17,704 (56.0%) of them had no distance problem to the health facility (Table 1).

**Table 1:** Sociodemographic characteristics of reproductive-age women in East Africa, DHS 2011–2021.

Variable	Category	Frequency	Percentage
Residence	Urban	7,485	23.7
	Rural	24,155	76.3
Maternal age in the year	15–24	11,687	36.9
	25–34	14,234	45.0
	35–49	5,719	18.1
Women’s education level	Unable to read and write	7,994	25.2
	Primarily education	15,854	50.1
	Secondary education	6,770	21.4
	Higher education	1,022	3.23
Marital status	Single	2,066	6.5
	Married	27,248	86.1
	Widowed	335	1.1
	Divorced	1,991	6.3
Religion	Orthodox	7,998	25.3
	Muslim	3,463	10.9
	Protestant	2,865	9.1
	Others	17,314	54.7
Wealth index	Poor	14,450	45.6
	Middle	6,183	19.5
	Rich	11,007	34.8
Media exposure	Yes	20,351	64.3
	No	11,289	35.7
Women’s occupation	Working	18,471	58.4
	Not working	13,169	41.6
Sex of household head	Male	23,216	73.4
	Female	8,424	26.6
Place of delivery	Home	7,176	22.7
	Health facility	24,464	77.3
Parity	Prim parous	7,232	22.9
	Multiparous	23,795	75.2
	Grand-multiparous	613	1.9
Number of ANC visit	No	3,258	10.3
	1–3	12,427	39.3
	Above 4	15,955	50.4
Birth interval	Short	7,555	23.8
	Normal	16,565	52.4
	Long	7,520	23.8
Husband education level	Unable to read and write	6,052	21.6
	Primarily education	13,575	48.4
	Secondary education	6,839	24.4
	Higher education	1,586	5.6
Distance to a health facility problem	Big problem	17,704	56.0
	Not a big problem	13,936	44.0
Mode of delivery	Normal	22,603	71.4
	Cesarean section	9,037	28.6
Sex of child	Male	15,825	50.0
	Female	15,815	50.0
Type of childbirth	Single birth	30,803	97.4
	Twin birth	837	2.6

### Pooled prevalence of breastfeeding initiation in East Africa

**Figure 2:**
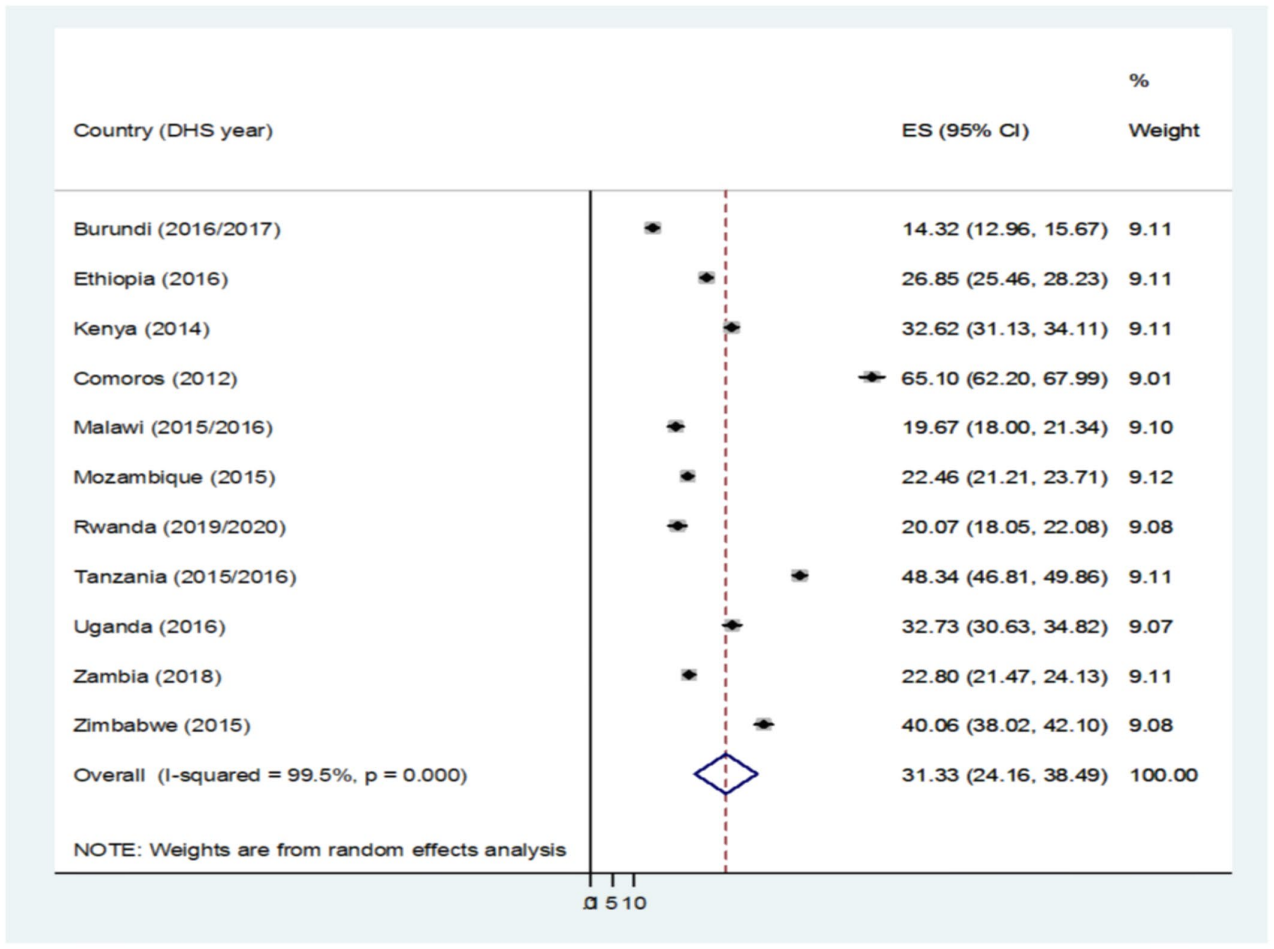
Pooled prevalence of breastfeeding initiation among reproductive-age women in East Africa.

The pooled prevalence of delayed breastfeeding initiation in East Africa was 31.33% with a 95% CI (24.16–38.49). The minimum prevalence of delayed breastfeeding initiation in Burundi (14.32%) with a 95% CI (12.96–15.67) and the maximum prevalence of delayed breastfeeding initiation in Comoros (65.10%) with a 95 %CI (62.20–67.99) (Figure 2).

### Machine learning analysis of breastfeeding initiation in East Africa

#### Balancing

**Figure 3:**
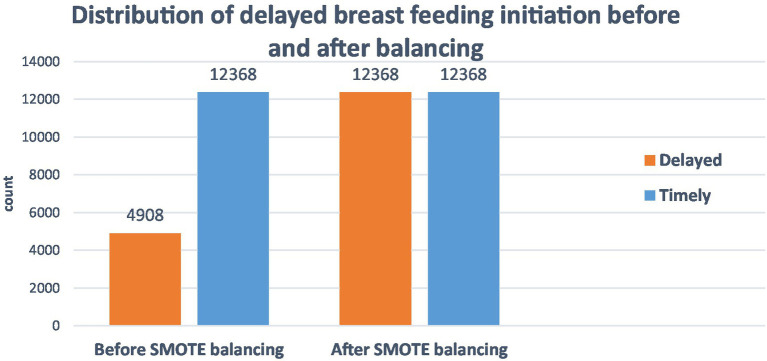
SMOTE balancing of home delivery after ANC visit among reproductive-age women in East Africa, DHS 2011–2021.

SMOTE oversampling generated 7,460 additional synthetic observations for the minority class (delayed breastfeeding initiation) to address the imbalance in the distribution. To establish symmetric distributions for both groups and dependable prediction models, the overall distribution of delayed breastfeeding initiation was adjusted from 12,368 timely BFI and 4,908 delayed BFI to 12,368 in each class of BFI (Figure 3).

### Model performance comparison

When classifiers were compared using stratified tenfold cross-validation and imbalanced training data, the Ada Boost machine emerged as the most successful model, exhibiting 73.08% accuracy and 61.24% area under the ROC curve. However, this result was deceptive since the outcome variable was unbalanced. As a result, after the training data were balanced using the SMOTE oversampling technique random forest was the best predictive model with an accuracy of 73.86% and an 80.68% area under the ROC curve (Table 2).

**Table 2:** Model comparison through cross-validation of training data.

Machine learning Models	Performance	Unbalanced (%)	Balanced (%)
Random forest (RF)	Accuracy	68.82	73.86
	AUC	59.12	80.68
Support vector machine(SVM)	Accuracy	72.83	66.26
	AUC	63.56	72.17
Logistic regression (LR)	Accuracy	72.85	63.79
	AUC	63.59	68.09
K-nearest neighbors (KNN)	Accuracy	68.18	67.44
	AUC	55.34	73.43
Ada Boost (AdB)	Accuracy	73.08	63.03
	AUC	61.24	67.47
Gaussian Naïve Bayes (GNB)	Accuracy	42.79	53.55
	AUC	60.74	63.98
Multi-layer perceptron (MLP)	Accuracy	70.42	66.57
	AUC	60.98	72.73
Decision tree (DT)	Accuracy	72.79	59.56
	AUC	62.52	63.99
Extreme gradient boosting (XG boost)	Accuracy	71.72	67.72
	AUC	62.09	73.53

On previously unseen test data, the prediction of delayed breastfeeding initiation was carried out following the selection of the best model (RF). Following random forest training on unbalanced training data, balanced data using default model parameters, and a comparison with an optimized model trained on balanced data, the prediction was made. After balanced and unbalanced data training for the random forest model, the prediction on unseen test data yielded an area under curve score of 0.78 and 0.82, respectively. Likewise, an AUC of 0.84 was predicted using a random forest with hyperparameter tuning.

### Hyperparameter tuning of random forest

Scikit-learn is not always the best solution for a given situation, even if it offers a set of reasonable default hyperparameters for every model. The number of decision trees in the forest, the number of features each tree considers when splitting a node, the minimum number of samples needed to split an internal node, the minimum number of samples needed to be at a leaf node, and the number of samples to draw from independent variables to train each tree were therefore optimized with 100 trials on a given search space using stratified 10-fold cross-validation to maximize the performance of the random forest (Table 3). Ultimately, using these adjusted hyperparameters on balanced training data, a random forest model was built using 10-fold cross-validation, producing an area under the curve of 0.82 and an accuracy of 83.8%.

**Table 3:** Default and optimally tuned hyperparameters of the Random Forest model.

Hyperparameter	Default	Optimal Value
Number of trees	100	200
Number of features considered for the best split	The square root of the number of features	0.17
The minimum number of samples required to split an internal node	2	2
The minimum number of samples required to be at a leaf node	1	1
Number of samples to draw from X to train each base estimator	None	0.97

### Important feature selection using random forest (RF)

The findings demonstrated that the top 10 features to predict delayed breastfeeding initiation were determined using the optimized random forest model with test data.

**Figure 4:**
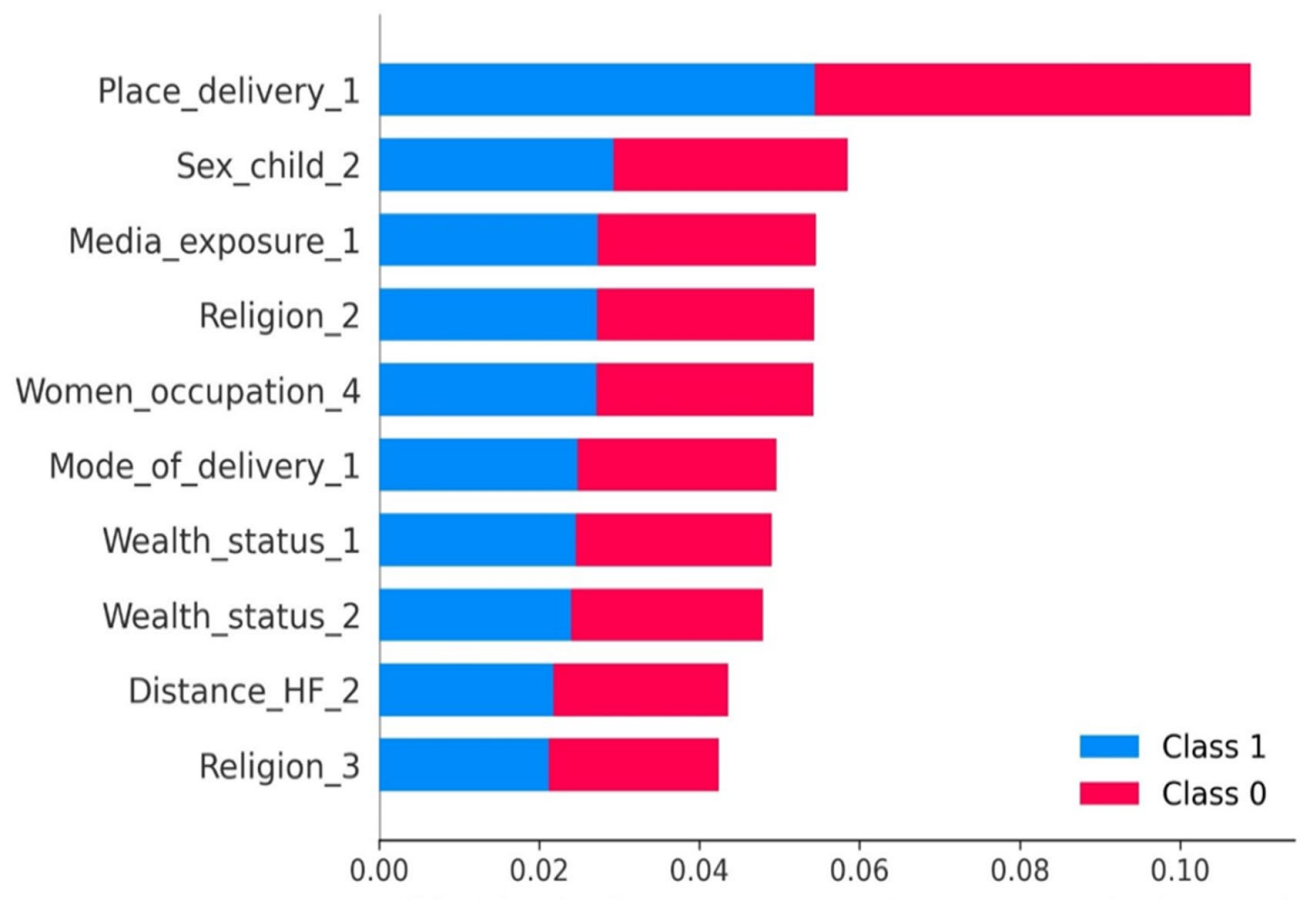
Feature importance plot of optimized random forest model. Note: birth delivery at health facility (place delivery-1), female children (sex_child_2), not exposed the media (media_exposure_1), Muslim religion follower (religion_2), being occupied (women_occupation-4), being delivered by cesarean section (mode_delivery-1), middle wealth status (wealth_status-1), rich wealth status (wealth_status-2), no problems of distance to health facility (distance_HF_2) and protestant religion follower (religion_3).

Based on their impact on the outcome variable prediction, the predictors are ranked in descending order, with characteristics with higher SHAP mean values having greater relevance. Thus, birth delivery at health facility (place delivery-1), female children (sex_child_2), not exposed the media (media_exposure_1), Muslim religion follower (religion_2), being occupied (women_occupation-4), being delivered by cesarean section (mode_delivery-1), middle wealth status (wealth_status-1), rich wealth status (wealth_status-2), no problems of distance to health facility (distance_HF_2) and protestant religion follower (religion_2), were also important predictors of breastfeeding initiation. The horizontal rectangles for each class are half-filled with the colors red and blue, as seen in the figure. This indicates that every characteristic has an equivalent effect on the categorization of instances involving timely (label = class 0) and delayed (label = class 1) breastfeeding initiation (Figure 4).

### Association rule mining

According to the Apriori algorithm, which generated seven rules, the following factors were most frequently associated with a high probability of delayed breastfeeding initiation: being a rural resident, home delivery, delivery by cesarean section, lack of media exposure, women aged between 35 and 49 years, and women who did not experience distance problems accessing health facilities.The top five association rules based on the probability of delayed breastfeeding initiation and their lift values are listed below.

**Rule 1. If ‘**place of delivery =2 (home delivery), mode of delivery = 1 (delivered by cesarean section), distance to health facility = 1 (had problem of distance to health facility), education status of husband = 1 (not attained formal education), number of ANC visit = 1 (had not ANC visit), birth interval = 1 (short birth interval)’, **Then** probability of delayed breastfeeding initiation is 94.4% (lift = 1.37).

**Rule 2. If ‘**women age = 3 (age between 35 and 49 years), residence = 1 (rural residence) mode of delivery (delivered by cesarean section), distance to health facility = 1 (had problem of distance to health facility), religion = 2 (Muslim), number of ANC visit = 2 (had 1–3 times ANC visit), place of delivery = 2 (home delivery)’, **Then** probability of delayed breastfeeding initiation is 93.3% (lift = 1.29).

**Rule 3. If ‘**women age = 3 (age between 35–49 years), place of delivery =2 (home delivery), residence = 1 (rural residence), mode of delivery = 1 (delivered by cesarean section), media exposure = 1 (not exposed on media), religion = 2 (Muslim)’, **Then** probability of delayed breastfeeding initiation is 92.5% (lift = 1.26).

**Rule 4. If ‘**residence = 1 (rural residence), mode of delivery = 1 (delivered by cesarean section), wealth status = 0 (poor wealth status), women occupation = 4 (had an occupation), number of ANC visit = 1 (had not ANC visit), birth interval = 1 (short birth interval)’, **Then** probability of delayed breastfeeding initiation is 92.3% (lift = 1.25).

**Rule 5. If ‘**women occupation = 4 (had an occupation), women age = 3 (age between 35 and 49 years), residence = 1 (rural residence), mode of delivery = 1 (delivered by cesarean section), religion = 2 (Muslim), distance to health facility = 1 (had the problem of distance to health facility)’, **Then** probability of delayed breastfeeding initiation is 92.3% (lift = 1.25).

## Discussion

This study aimed to assess the effectiveness of a machine learning algorithm to pinpoint significant factors associated with delayed breastfeeding initiation in East Africa. Using unbalanced training data, the Extreme Gradient Boost (XGb) model classifier outperformed other classifiers in the early stages of predictive modeling. On balanced training data, Random Forest (RF) performed better than other model classifiers in the second stage of model prediction. Fitting the random forest prediction model to test data showed that it was the most effective.

According to our analysis, the prevalence of delayed initiation of breastfeeding in East Africa was 31.33%, and this finding fairly agrees with the findings of the studies conducted in Colombia (34.4%) (1), Bangladesh (30.4%) (2), Nepal (33%) (3), Pakistan (32%) (4), Ghana (28%) (5), Sudan (31%) (6), Tanzania (30%) (7), and Dire Dawa (29.1%) (8).

However, the study findingwas slightly higher than the prevalence reported in EDHS (26.7%) (9), as well as the findings of previous studies conducted in Haiti (24.1%) (10), Debre Tabor (23.2%) (11), and Dembecha (26.9%) (12). On the contrary, it was significantly lower than the findings of the studies conducted in Jashore District of Bangladesh (53.7%) (13), Western Nepal (57.8%) (14), India (63.6%) (15), Saudi Arabia (88.6%) (16), Mizan-Aman (64.5%) (17), Debre Markos (75%) (18), and Dabat (56.1%) (19). These discrepancies may be attributed to variations in the socioeconomic backgrounds of the mothers studied. Higher maternal education levels generally lead to better knowledge and practices regarding early breastfeeding. Cultural norms significantly impact breastfeeding behaviors, with cultures supporting immediate breastfeeding showing lower rates of delay. Religious beliefs can also influence breastfeeding practices and timing. Income levels affect access to healthcare services, with higher income providing better resources and support for early breastfeeding initiation (19–21).

The association rule mining result showed that home delivery, delivered by cesarean section, poor wealth status, poor access to media outlets, women aged between 35 and 49 years, and women who had distance problems to access health facilities were associated with delayed breastfeeding initiation in East Africa.

This study found that giving birth in healthcare facilities decreases the odds of delaying the initiation of breastfeeding among mothers in East Africa, and a similar conclusion was reached by those studies conducted in Indonesia (22), Southern Ethiopia (23), East Gojjam (24), and Dire Dawa, Ethiopia (8). These similarities may stem from the fact that mothers who give birth in health facilities are more likely to receive health information, nutrition education, and guidance from well-trained health professionals. These professionals emphasize the importance of initiating breastfeeding within the first hour of delivery, which promotes a stronger bond between mother and child, improves newborn breathing patterns, ensures the infant receives colostrum, and reduces the risks of hypothermia and postpartum hemorrhage (25–27).

Conversely, mothers who gave birth at home might have been influenced by the culturally shaped perspectives of traditional birth attendants, family members, or the broader community. These views may not always support the health of newborns and their mothers. For example, in some cultures, newborns are given foods like butter or herbal concoctions before breastfeeding to prepare their gastrointestinal tract for future meals, rather than initiating breastfeeding immediately (28). In some cultures, the first breast milk, or colostrum, is discarded because it is perceived as unclean or undesirable (29, 30).

However, this may not be always the case. According to some recent studies, health facility delivery was identified as one of the risk factors for delaying the initiation of breastfeeding (21, 31, 32). This might attributed to the aggressive marketing tactics of breast milk substitute companies. These tactics often involve providing free samples of infant formula to women delivering in healthcare facilities and actively persuading healthcare providers to recommend these substitutes to mothers before breastfeeding is initiated (33, 34).

In addition, our study revealed that mothers who had limited access to media outlets were more likely to delay the initiation of breastfeeding, and this find was supported by another study conducted in Dembecha town of Ethiopia (12), This could be because media outlets are a key method for disseminating health-related information to expectant women and their families, especially in developing countries where access to healthcare facilities may be limited. Media, including television, radio, and mobile devices, effectively reaches a broad audience. Consequently, both governmental and non-governmental agencies in these regions often use media to promote optimal breastfeeding practices. Among East African women, access to media outlets may have enhanced their receipt of health information, improving their knowledge and attitudes toward optimal breastfeeding practices and, consequently, their likelihood of initiating breastfeeding early.

Furthermore, women who were in the middle- and high-income categories and those who had jobs were less likely to delay the initiation of breastfeeding. Likewise, a review that assessed the predictors of early initiation of breastfeeding among Asian mothers reached at same conclusions as our study (35). The possible justification for such similar findings might be women in middle- and high-income categories, as well as those who were employed, are more likely to be financially secure, and such security often grants mothers access to high-quality education and healthcare services. As a result of this access, those women who were in middle- and high-income categories, as well as those who were employed might have a better understanding of the importance associated with initiating breastfeeding early. In addition, financially secure women are more likely to possess expensive but important means of obtaining health-related information, such as TV and smartphones.

However, financially insecure women are often reliant on their partners, families, and spouses for financial support. As a result, they might be subjected to low-quality and overcrowded public healthcare facilities, demotivating them from fully attending antenatal and postnatal healthcare services. Furthermore, due to financial constraints, women in the low-income category might not be able to afford high-quality education and expensive gadgets such as cable TV and smartphones. This might force them to rely on information they acquire from other individuals (neighbors, friends, colleagues, etc.).

Moreover, our study found lesser odds of delaying the initiation of breastfeeding among those women who were between the age of 35–49. Likewise, studies conducted in Bangladesh and Dire Dawa found lesser odds of delaying the initiation of breastfeeding among older women (8, 36). This finding may be due to older women having had multiple pregnancies, which increases their likelihood of visiting healthcare facilities more frequently. These repeated visits provide more opportunities for receiving nutrition education, which can enhance their knowledge and improve their attitudes toward optimal infant and young child feeding practices.

In addition to the previously mentioned predictors of delayed initiation of breastfeeding, our study found that women who did not face distance-related challenges to the nearest healthcare facilities were less likely to delay breastfeeding initiation. This could be because proximity to healthcare facilities serves as both an advantage and a barrier. In developing countries, where transportation services may be inadequate, women often travel long distances to access healthcare. This journey poses risks such as kidnapping, assault, or even death. To avoid these dangers, some women may choose to stay at home and rely on traditional birth attendants or community members instead of seeking professional medical care.

This study found that women who lived in rural areas had an increased chance of delaying the initiation of breastfeeding. This finding was in line with previous studies in India (49), Bangladesh (50), and Ethiopia (35). This might be because women in urban and rural areas have differing access to and utilization of healthcare and educational resources. Women in rural areas often have less access to essential healthcare services and high-quality education compared to their urban counterparts. As a result, especially in underdeveloped nations, rural women may be less informed about optimal breastfeeding techniques (51).

Contrarily, studies done in Australia (52) and Ethiopia (53) found an improvement in the rate of early initiation of breastfeeding among women residing in rural areas compared to those in urban areas. This could be attributed to the evolving nature of work that women in urban areas are engaged in, which often requires longer hours, leaving them with less time for child-rearing, another explanation might be our study utilized a larger sample size, providing more robust and representative data, which enhances the reliability of the findings. Additionally, the study period may differ, affecting how changes over time influence breastfeeding practices. These methodological differences, including sample size and the duration of the study, can significantly impact the findings in various settings.

Finally, women who had delivered through cesarean section were more likely to delay the initiation of breastfeeding. Supporting this finding, studies conducted in Bangladesh, India, El-Minia University Hospital, and Indonesia reached the same conclusion (2, 15, 22, 37). This finding might arise from the fact that, mothers who undergo cesarean section deliveries often experience significant postoperative pain and fatigue, which can hinder their ability to initiate breastfeeding promptly. In developing countries, inadequate pain management practices further exacerbate this issue, making it difficult for mothers to engage in skin to skin contact and early breastfeeding. Another reason might be the separation of babies from their mothers following an operation, which can prevent mothers from being near their babies and breastfeeding them as needed.

### Limitations and strengths of the study

The study’s main strengths include the use of a large sample size and nationally representative data, as well as the application of an advanced statistical method, specifically a machine learning approach. Association rule mining was employed to identify significant factors and patterns contributing to delays in breastfeeding initiation.

However, the study has limitations. The reliance on self-reported data from the DHS survey may introduce bias due to potential inaccuracies in recalling past events. This study also only reveal correlations and not the underlying causal relationships. Moreover, our study is the lack of birth-related data, such as maternal and infant health conditions, Apgar score, gestational age, and birth weight, which could potentially limit the study’s generalizability of the findings. Future studies could benefit from exploring additional predictive variables that can be identified and measured before birth or shortly after delivery.

## Conclusion

The prevalence of delayed breastfeeding initiation among women with children less than 2 months of age in East Africa was high. This study employed nine machine learning algorithms, to predict delayed breastfeeding initiation and pinpoint associated factors among women with less than 2 months of childhood in East Africa according to a recent DHS dataset. From the included models, random forest was the best model to predict delayed breastfeeding initiation.

The association rule mining findings showed that, home delivery, delivered by cesarean section, poor wealth status, poor access to media outlets, women aged between 35 and 49 years, and women who had distance problems accessing health facilities were associated with delayed breastfeeding initiation in East Africa. Policymakers and stakeholders pay attention to the significant factors and we recommend targeted interventions to improve healthcare accessibility, enhance media outreach, and support women of lower socioeconomic status. These measures can encourage timely breastfeeding initiation and address the identified factors contributing to delays across the region in East Africa.

## Data Availability

The dataset used in our study can be accessed through the DHS Program. For ease of access, please visit https://dhsprogram.com/data/dataset_admin/. After visiting this link, you will need to register to access and download the specific datasets used in our study. The DHS Program allows the distribution of its datasets for legitimate academic research, provided users adhere to specific conditions. These include using the data solely for the registered research, requesting permission for any new use, and not sharing the datasets with others without written consent. Researchers must keep the data secure, treat it as confidential, avoid identifying individuals or communities, and ensure that the data is not used for commercial purposes or redistributed in any form. Further inquiries can be directed to the corresponding author.
